# Risk assessment and source apportionment of trace elements in multiple compartments in the lower reach of the Jinsha River, China

**DOI:** 10.1038/s41598-021-99626-w

**Published:** 2021-10-08

**Authors:** Wenyan He, Fei Li, Jiang Yu, Min Chen, Yun Deng, Jia Li, Xiliang Tang, Zhuoyu Chen, Zhongluan Yan

**Affiliations:** 1 State Key Laboratory of Hydraulics and Mountain River Engineering, College of Water Resource and Hydropower, Sichuan University, No. 24 South Section 1, Yihuan Road, Chengdu, 610065 China; 2 Changjiang Water Resources Protection Institute, Wuhan, 430051 China; 3 China Three Gorges Projects Development Co., Ltd,, Chengdu, 610000 China

**Keywords:** Environmental sciences, Environmental chemistry, Environmental impact

## Abstract

Studies on trace element (TE) pollution in abiotic matrices have typically focused on water, sediment, and soil, either separately or in pairs. The importance of multi-media connectivity has been ignored. This study analyzed the concentrations of 6 TEs in three connected environmental compartments of a 28-km section of the lower reach of the Jinsha River. The ecological risk posed by TEs was higher in soil than in sediment. The contribution of exposure pathways to human health risk were ranked as ingestion > dermal contact > inhalation. An improved regional environmental risk index (*RERI*) method was then developed to evaluate the comprehensive risk on both ecology and human health caused by TEs. The average *RERI* value was generally higher in the wet season (0.42) than in the dry (0.41) and dry-to-wet transition seasons (0.08) because of the combined effects of the high TE concentrations in riparian soil and the long exposure time. Source apportionment indicated that industrial activities, weathering of parent rock, and agricultural activities were possible sources of TEs in this region. The methods and results of this study could inform local environmental management and provide references for similar cases wherein multiple compartments of river systems should be considered.

## Introduction

After the rapid development of industrialization and urbanization, the health of river ecosystems has gradually received much attention^1^. Rivers are highly dynamic and vulnerable to the effects of the surrounding environment^2^, as they serve as important catchment areas for municipal and industrial sewage. Trace elements (TEs) in the riparian zone enter the aquatic environment when the water level fluctuates, during rainfall events, or when floods occur, which can temporarily alter the water quality^3^. Sediment, which serves as a large sink of TEs at the bottom of rivers, plays a vital role in TE migration and circulation^4^. TEs in the sediment may be remobilized and released to surface water via hydrodynamic disturbance (e.g., reservoir drainage, wind-driven currents in shallow lakes, etc.) or bioturbation, leading to an unexpected change in water quality^5^. The connectivity among water, riparian soil, and sediment provides a channel for chemical transport, so the risk of TEs in one environmental compartment could shift to another. Therefore, the migration and transformation of TEs in multiple compartments of ecosystems has attracted more attention in recent years^6–8^. In addition, for river ecosystems affected by industry and regulating reservoirs, the material exchange process in multiple compartments may be more frequent; thus, a full-scale study is needed.

The Jinsha River, which is the upper course of the Yangtze River, originates from the Tibetan Plateau regarded as an eco-safety barrier in China and Asia. However, one of the largest vanadium titanomagnetite mining bases in China is located near the initial section of the lower reach of the Jinsha River (LRJR)^9^, posing a major environmental threat to downstream water quality and riparian ecosystem health. The ecological characteristics of the area accelerate this threat: the Jinsha River dry-hot valley (DHV) is considered an ecologically fragile zone susceptible to critical soil erosion^10^. Tang^11^ demonstrated that the total soil erosion from the upper Yangtze River is approximately 2.2 × 10^9^ t/yr. Terrestrial pollutants enter the river ecosystem with soil erosion. Moreover, four large cascade dams have been constructed or are under construction in the LRJR, which may intensify this threat because the impoundment of reservoirs would slow the water speed, diminish the water self-purification capacity, and lead to the retention of contaminants in sediments^12^. There are concerns about whether TEs from uplands will be trapped in cascade reservoirs. As a result, it was necessary to assess the current pollution status of the potential risk area before impoundment.

Previous research conducted near the vanadium titanomagnetite mining area in the LRJR basin demonstrated that V, Cu, Zn, As, Cd, and Pb were typical pollutants in the soil^13, 14^. Several methods and tools have been developed to assess the ecological and human health risks of these TEs, such as enrichment factor analysis^4^, the geo-accumulation index^15, 16^, the pollution load index^17^, the potential ecological risk index^18^, and the USEPA^19^ human health risk assessment protocols. However, the commonly used environmental risk assessment methods evaluate ecological risk and human health risk independently. It is essential to develop a method to assess the overall risk. Furthermore, much research has sought to identify possible sources of TE pollution with the aim of controlling risk. The isotope ratio method is based on the isotope mass conservation principle; however, it is suitable only for certain TEs, such as Pb, Zn, Cu, Cd, and Hg^20^, and it is expensive, which limits its application. Traditional multivariate statistical methods, including factor analysis, principal component analysis, and cluster analysis^21–23^, have been widely used to qualitatively determine the source of specific TEs by identifying TEs with similar distribution characteristics.

Therefore, field surveys were conducted in the LRJR section affected by industrial activities before the operation of the downstream reservoir to clarify the status of Cu, Zn, V, As, Cd, and Pb. The present study aimed to (i) evaluate the TE pollution status of three connected environmental media, (ii) assess the ecological and human health risks, (iii) develop an improved regional environmental risk assessment method, and (iv) identify the TE source(s) in this river ecosystem. The research results could be a reference for environmental management in a similar ecologically fragile and intense industry zone and provide theoretical support for further policy-making to protect river ecosystems.

## Materials and methods

### Study area

**Figure 1:**
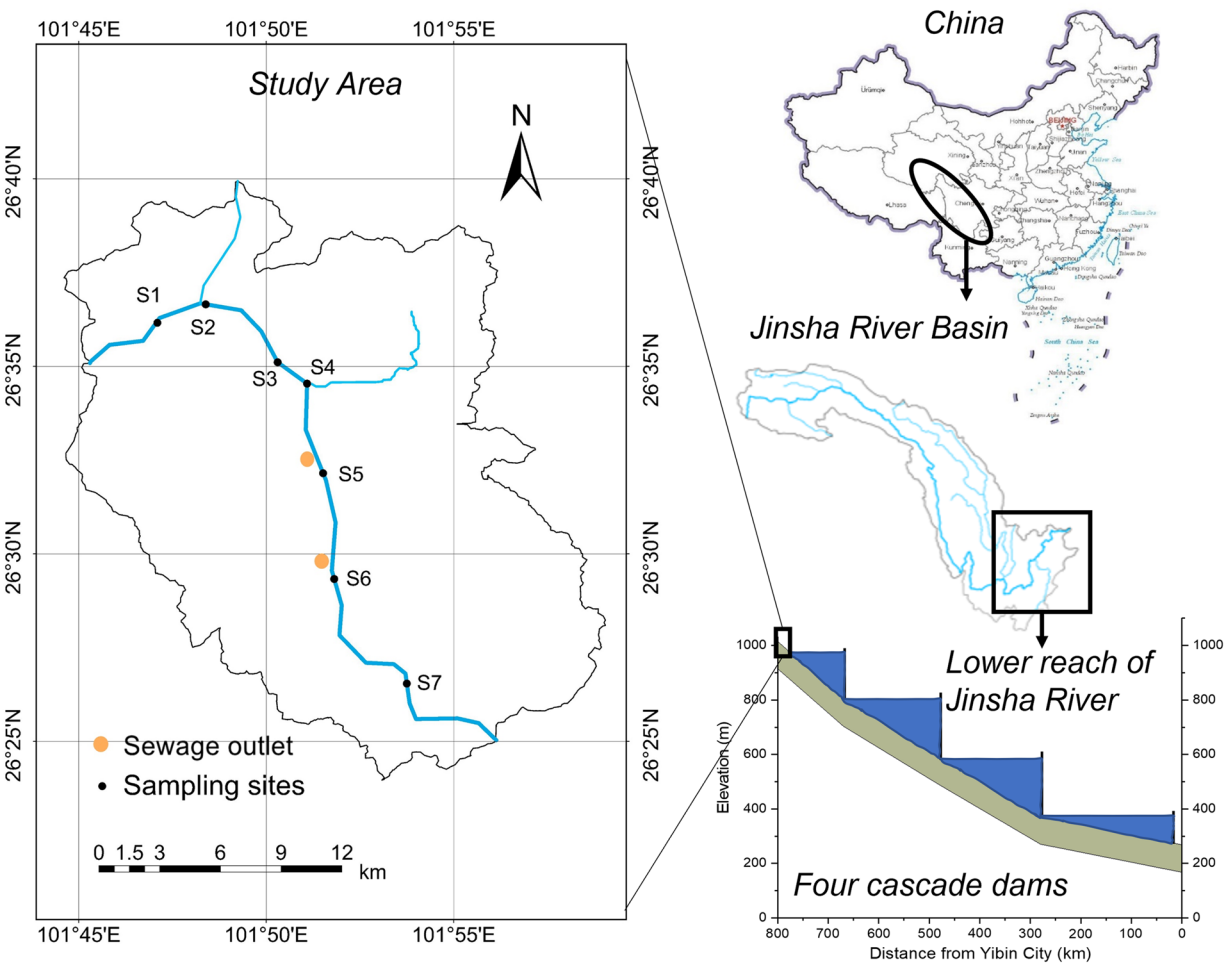
Location of the study area and distribution of sampling sites. The map of Jinsha River Basin and study area were generated using the ArcGIS Desktop (ESRI, Inc, Version 10.7, URL:https://desktop.arcgis.com/zh-cn/). The map of China was downloaded from Standard Map Services, which is an free access map database provided by the Ministry of Natural Resources of the People’ Republic of China (URL: http://bzdt.ch.mnr.gov.cn/browse.html?picId=%224o28b0625501ad13015501ad2bfc0290%22, reference number: GS(2019)1651).

The study area (26° 25′–26° 40′ N and 101° 45′–101° 55′ E) is the first 28-km section of the LRJR (Fig. 1). The river valley is a typical DHV with a high temperature (mean annual temperature of 18–23 °C) and an unequal distribution of precipitation (mean annual precipitation of 800–1200 mm and evaporation of 2500–4000 mm). A large vanadium titanomagnetite mining region and its accompanying downstream industries are located near the study area, and it is the main pollutant source in the Jinsha River. The gross product of this area in 2012 was 43,028.71 million CNY, with industrial output accounting for 99.55%^24^. Additionally, our study area is located in the upstream boundary of these four cascade large reservoirs.

### Sample collection and analysis

Field surveys were conducted in March, April, and July 2018, corresponding to the dry, dry-to-wet transition, and wet seasons, respectively^25, 26^. We collected water, soil, and sediment samples from 7 sites. Among these sampling sites, S2 and S4 were near tributary inlets, S5 was near a municipal sewage outlet, and S6 was near an industrial sewage outlet (Fig. 1). Forty-five samples were collected, including 21 water samples, 18 soil samples, and 6 sediment samples. Water samples were collected from the midstream section of the watercourse. In the field, water samples were acidified with 1 M HNO_3_ to achieve a pH value under 2.00 and stored in polyethylene bottles. Sediment samples were collected from a location vertically below the water samples at a surface depth of 0–30 cm. Due to the high runoff of the Jinsha River, sediment samples could not be collected in the dry-to-wet transition and wet seasons. Soil samples were collected at the longitudinal extension line of the water sampling point intersecting the floodplain at a depth of 0–20 cm. Sediment and soil samples were collected with a stainless grab or shovel, peeled off the surface layer and then discarded, and the residue was placed in polypropylene bags, which were sealed for storage. In addition, these three types of samples were taken in the same place in each season. The samples were transferred, pretreated, and analyzed following the national standard of China^27^. Briefly, water samples were totally digested by HNO_3_ (1 mL HNO_3_) assisted by microwaves. Sediments and soils were ground by a mortar and pestle through a 0.075-mm nylon sieve and then completely digested by HCl-HNO_3_-HF-H_2_O_2_ (i.e., 1 mL HCl, 4 mL HNO_3_, 1 mL HF, and 1 mL H_2_O_2_) assisted by microwaves. All samples were digested in duplicate. The concentrations of Cu, Zn, V, As, Cd, and Pb in the digestion solution were measured by inductively coupled plasma-mass spectrometry (ICP-MS, NexION 350X, Perkin Elmer, USA) with detection limits of 5, 5, 2, 0.07, 5, and 20 μg/L, respectively. Reagent and procedural blanks were measured in parallel with the samples. Each calibration curve was evaluated by analyzing the quality control standards before, during, and after the analyses of each set of samples. The same treatment was performed on certified sediment (GSD-8) and soil (GSS-8) certified reference materials during the digestion and analysis procedures to validate the method's accuracy and for quality control. All experimental vessels were soaked in 10% HNO_3_ before use.

### Research methodology

As the studied reach is located in Panzhihua, which is the first city on the upper reaches of the Yangtze River, both the ecological risk and human health risk should be considered to comprehensively evaluate the environmental risk posed by TEs in the study area.

#### Potential ecological risk index

The Hakanson potential ecological risk index was developed to evaluate the potential effects of TEs in sediment on the ecosystem^18^, and its application has been expanded to soil^28^. This method introduces the biological toxicity coefficient (*T*_*i*_) of TE *i*, which fully considers the distinctive potential effects of different TEs on the ecosystem. The potential ecological risk index is defined mathematically as follows:

1 $$RI_{i} = (T_{i} \times C_{i} {)}/S_{i}$$

2 $$RI = \sum {RI_{i} }$$

where *C*_*i*_ is the actual concentration of TE *i*, mg/kg, *S*_*i*_ is the background value of element *i* in the soil, *RI*_*i*_ is the potential ecological risk index of a given TE *i*, *RI* is the comprehensive potential ecological risk index of the detected TE, and *T*_*i*_ is the biological toxicity coefficient of TE *i*. The *RI*_*i*_ and *RI* values were divided into 5 categories of risk grade based on their toxicity coefficients, as shown in Supplementary Table S1.

#### Human health risk assessment

An exposure route is a connection between a receptor and a contaminated source^19^. People are exposed to TEs by ingestion, inhalation, or dermal contact with substances that contain TEs^15, 21, 29^. Individuals are exposed to TEs in water by ingestion and via skin adsorption when washing, showering, and swimming^19^. Human are exposure to TEs in soil occurs through the intentional or inadvertent non-dietary ingestion of soil on hands or foods via hand-to-mouth or object-to-mouth activity, by inhaling contaminated air, and through skin contact with contaminated soil^19^. The common sediment exposure route is through wading; however, the water depth of the study area is too deep (above 2 m) for wading, allowing us to ignore the human health risk posed by TEs in sediment. The relative contribution of each exposure route to the chronic daily intake (*CDI*) can be calculated by Eqs. (3)–(5):

3 $$CDI_{ing,i} = \left( {C_{i} \times IR_{ing} \times EF \times ED \times CF} \right) \div \left( {BW \times AT} \right)$$

4 $$CDI_{derm,i} = \left( {C_{i} \times SA \times AF \times ABS \times EF \times ED \times CF} \right) \div \left( {BW \times AT} \right)$$

5 $$CDI_{inh,i} = \left( {C_{i} \times IR_{inh} \times EF \times ED} \right) \div \left( {PEF \times BW \times AT} \right)$$

where *CDI*_*ing, i*_, *CDI*_*derm, i*_, and *CDI*_*ing, i*_ represent the chronic daily intake of TE *i* by ingestion, dermal contact, and inhalation, respectively, mg/(kg·d); *IR*_*ing*_ is the adult daily oral ingestion rate of soils (100 mg/d) and water (2.5 L/d)^21^; and *EF *denotes the exposure frequency to both water and soil. The *EF* value recommended by the USEPA^30^ is 350 d/yr; however, to understand the *CDI* variance in different flow seasons, we chose 150, 60, and 150 d/yr to represent the dry, dry-to-wet transition, and wet seasons, respectively, based on local hydrologic features^25, 26^. Additionally, *ED* is the exposure duration of adults (24 years); *CF* is the average conversion factor (10^−6^ kg/mg)^30^; *BW* is the average body weight of adults (56.8 kg)^30^; *AT* is the average time of a noncarcinogenic effect (35 years, 12,775 d) or carcinogenic effect (70 years, 25,550 d)^15^; *SA* is the adult surface area of exposure affected by dermal contact (1530 cm^2^)^15^; *AF* is the adherence factor of the skin and soil (0.49 mg/cm^2^)^31^; *ABS* is a dermal adsorption factor (0.001)^29^; *IR*_*inh*_ is the adult daily inhalation rate of soil (20 m^3^/d)^15^ and water (0.0017 m^3^/d)^32^; and *PEF* is the particle emission factor (1.36 × 10^9^ m^3^/kg)^17^.

The human health risk of TEs was divided into noncarcinogenic and carcinogenic risks. The noncarcinogenic risk estimates the risk after an exposure dose exceeds a certain value, which varies depending on the TE species and the exposure route^32^. The hazard index (*HI*_*i*_) is used for exposure to a single TE and can be calculated by Eq. (6):

6 $$HI_{i} = CDI_{ing,i} \div RfD_{{_{ing,i} }} { + }CDI_{derm,i} \div RfD_{derm,i} { + }CDI_{inh,i} \div RfD_{inh,i}$$

where *RfD*_*ing, i*_, *RfD*_*derm, i*_, and *RfD*_*inh, i*_ are the reference doses of the ingestion, dermal contact, and inhalation exposure routes, respectively, mg/(kg·d). The *RfD* values of different TE exposure pathways are given in Supplementary Table S2.

The cumulative hazard index (*HI*) was introduced to describe the *HI*_*i*_ of all TE species and was calculated by Eq. (7).

7 $$HI{ = }\sum {HI_{i} }$$

When *HI* < 1, the noncarcinogenic risk is low; when 1 < *HI* < 4, the noncarcinogenic risk is acceptable; and when *HI* > 4, the noncarcinogenic risk is high^30^.

Carcinogenic risk can be defined as the probability of individuals developing lifelong cancer caused by chronic exposure to carcinogenic species^32^. The carcinogenic risk of an individual TE (*CR*_*i*_) was calculated using Eq. (8):

8 $$CR_{i} = CDI_{ing,i} \times SF_{ing,i} + CDI_{derm,i} \times SF_{{{\text{de}}rm,i}} + CDI_{inh,i} \times SF_{{_{inh,i} }}$$

where *SF*_*ing, i*_, *SF*_*derm, i*_, and *SF*_*inh, i*_ are the carcinogenic slope factors of the ingestion, dermal contact, and inhalation exposure routes, respectively, mg/(kg·d). The *SF* values of different TE exposure pathways are given in Supplementary Table S2.

The cumulative carcinogenic risk (*CR*) was introduced to describe the *CR*_*i*_ of all TE species and was calculated by Eq. (9).

9 $$CR{ = }\sum {CR_{i} }$$

When *CR* > 10^−4^, the carcinogenic risk is high; when 10^−6^ < *CR* < 10^−4^, the carcinogenic risk is low; and when *CR* < 10^−6^, no significant carcinogenic risk to humans exists^15, 21^.

#### Regional environmental risk assessment

An improved method called the regional environmental risk index (*RERI*) method was developed to quantitatively calculate the overall risk to both local ecology and human health posed by TEs. The min–max normalization method was used to map the data on ecological risk results and human health risk results in a range from 0 to 1. This method allowed us to compare the values at the same order of magnitude. The weights assigned to ecological risk and human health risk were obtained by the analytical hierarchical process. The regional environmental risk was then calculated by Eq. (10):

10 $$RERI{ = }\frac{{RI - RI_{{{\text{min}}}} }}{{RI_{{{\text{max}}}} - RI_{{{\text{min}}}} }} \times w_{1} + \frac{{\left( {HI + CR} \right) - \left( {HI + CR} \right)_{{{\text{min}}}} }}{{\left( {HI + CR} \right)_{{{\text{max}}}} - \left( {HI + CR} \right)_{{{\text{min}}}} }} \times w{}_{2}$$

where *w*_1_ and *w*_2_ are the weights of ecological risk and human health risk, respectively. *RERI* varies from 0–1 theoretically, where *RERI* < 0.25, 0.25 < *RERI* ≤ 0.5, 0.5 < *RERI* ≤ 0.75, and 0.75 < *RERI* ≤ 1.00 represent low, considerable, high, and severe risk levels, respectively.

### Statistical analysis

When the results were under the detection limit of the analysis method, we selected 1/2 of the detection limit to perform the statistical analysis^33^. All data were normally distributed. The coefficients of variation of the TE concentrations in different media were calculated to compare their degree of dispersion. Pearson correlation analysis and principal component analysis (PCA) were conducted to evaluate the relationships between different soil TEs^3, 15, 28^. All statistical analyses were performed using Origin 2020 (Student Version, Origin Lab Corp., USA).

## Results and discussion

### TE concentrations in water, soil, and sediment

The basic descriptive statistics of the TE concentration in water, soil, and sediment are shown in Table 1. The mean concentrations of TEs in water of the LRJR followed the order Zn > Cu > V > As, among which V had the largest variability in concentration, with a coefficient of variation (*CV*) of 75.21%. The concentrations of Cu, Zn, and As were below the first-grade state standard limit (10, 50, and 50 μg/L) of China^34^, which is the highest standard criterion. These TEs had mean values of 2.76, 9.38, and 1.75 μg/L, respectively. The V concentrations in water varied from N.D. to 6.00 μg/L, which were all below the acceptable threshold (50 μg/L)^34^, although a national-level vanadium-titanium high-tech industrial development zone was located near the study area. The concentrations of Cd and Pb in water were below the detection limits (5 μg/L for Cd and 20 μg/L for Pb), meeting the Chinese third-grade state standard limitations (5 and 50 μg/L, respectively)^34^. The TE concentrations observed in the LRJR and in other selected areas around the world are displayed in Table 2 for comparison. Similar to LRJR, Lake Pontchartrain (USA), the Ganga River (India), and Kharg Coral Island (Iran) are located near dense industrial areas. The TE concentrations of Kharg Coral Island seawater are of the same order of magnitude as those in our study area^6^. The area with the highest reported water Zn concentration (528 μg/L) is Lake Pontchartrain^35^. However, the water concentrations of Cu, Zn, Cd, and Pb in the Ganga River were slightly higher than that those observed in other areas^36^.

**Table 1:** Occurrence of trace elements in water, soil, and sediment in the study area.

	Cu	Zn	V	As	Cd	Pb
**Water (n = 42)**						
Min	N.D	N.D	N.D	0.70	N.D	N.D
Max	8.00	26.00	6.00	2.70	N.D	N.D
Mean	2.76	9.38	2.33	1.78	–	–
SD	1.17	5.35	1.75	0.51	–	–
CV (%)	42.41	56.98	75.21	28.77	–	–
**Soil (n = 36)**						
Min	28.60	61.00	138.00	1.09	0.10	4.16
Max	161.00	232.00	475.00	26.40	0.68	46.20
Mean	57.13	102.14	286.11	8.89	0.26	18.60
SD	28.72	39.22	89.77	7.63	0.13	11.04
CV (%)	50.27	38.40	31.38	85.74	50.89	59.38
**Sediment (n = 12)**						
Min	32.30	51.90	222.00	1.42	0.17	5.70
Max	54.00	97.80	333.00	4.98	0.34	20.50
Mean	37.82	74.43	274.83	3.55	0.24	11.75
SD	7.48	16.34	33.35	1.32	0.06	5.23
CV (%)	19.77	21.95	12.14	37.08	25.93	44.55
BV^a^	45	95	130	13	0.3	20
*T*^b^	5	1	2	10	30	5

*BV* Background value of soils in the study area, *T* Biological toxicity coefficient of element.

^a^Data are from Tuo et al.^37^.

^b^Data are from Hakanson^18^ and Xu, et al.^38^.

**Table 2:** Trace element levels in water, soil, and sediment from different countries/regions.

Location	Cu	Zn	V	As	Cd	Pb	Sample type	Sampling time
Lower reach of Jinsha River, China	N.D.–8.00	N.D.–26.00	N.D.–6.00	0.70–2.70	–	–	Water	Mar., Apr., and Jul. 2018
	28.60–161.00	61.00–232.00	138.00–475.00	1.09–26.40	0.10–0.68	4.16–46.20	Soil	
	32.30–54.00	51.9–97.80	222.00–333.00	1.42–4.98	0.17–0.34	5.70–20.50	Sediment	
Jinsha River, China^39^	10.30–247.61	2.90–129.97	–	2.22–30.53	0.05–0.73	4.08–44.04	Sediment	May. 2017
Yellow River area, China^28, 40, 41^	0.73–3.50	0.04–4.19	–	2.35–6.42	0.02–0.05	0.02–0.38	Water	Jul. 2011
	12.23–67.98	68.93–117.76	–	5.3–16.72	0.21–1.07	17.87–63.72	Soil	–
	14.1–30.3	39.3–74.6	–	–	0.1–0.3	–	Sediment	May. 2014
Yangzte River, China^42^	11.3–920	34.1–1050	–	4.7–278	0.09–29.39	15.9–535	Sediment	Nov. 2007–Jan. 2008
Yangzte River area, China^43^	17.31–72.50	47.87–332.40	–	–	0.02–2.72	12.14–83.85	Soil	–
Lake Pontchartrain, USA^35^	2.15–4.2	24.7–528	–	–	0.05–0.35	–	Water	Feb. and Jun. 2009
Ganga River area, India^36^	19.42–43.72	31.73–71.37	–	–	11.41–39.24	80.55–134.8	Water	Jul. 2012–Jun. 2013
Kharg Coral Island, Iran^6^	0.69–4.70	0.20–1.05	2.18–5.57	0.04–1.16	0.41–1.11	0.13–1.88	Seawater	Jan. 2015
	13.11–26.51	7.28–30.81	9.55–38.50	0.46–3.75	0.28–2.05	0.38–1.67	Sediment	

The unit of trace element concentration is μg/L for water samples and mg/kg for soil/sediment samples.

The concentration ranges of each TE detected in the soils of the LRJR were as follows: 28.60–161.00 mg/kg for Cu, 61.00–232.00 mg/kg for Zn, 138.00–475.00 mg/kg for V, N.D.–26.40 mg/kg for As, 0.10–0.68 mg/kg for Cd, and 4.16–46.20 mg/kg for Pb. The *CV* of As was extremely high (85.74%). The mean concentration values of Cu, Zn, and V were 1.27, 1.07, and 2.20 times the background values of Panzhihua soil, respectively, and those of As, Cd, and Pb were below this background value. The maximum concentration for five of the six TEs occurred at S6; this result was closely related to the industrial activity of the national-level vanadium-titanium high-tech industrial development zone around S6. The V products of this industrial park account for 60% and 20% of the domestic and international markets, respectively^44^. Furthermore, a previous study reported that this area has a high soil V content^9^. The soil concentrations of Zn, Cd, and Pb reported for the lower reach of the Yangtze River^43^ are higher than those in the LRJR (Table 2) due to intensive human and industrial activities. The maximum soil concentrations of Cu, Zn, and As observed in the LRJR are higher than those in the Yellow River area recorded by Liu and Liu^40^.

The concentration ranges of Cu, Zn, V, As, Cd, and Pb detected in the sediments of the LRJR were 32.30–54.00 mg/kg, 51.90–97.80 mg/kg, 222.00–333.00 mg/kg, N.D.–4.98 mg/kg, 0.17–0.34 mg/kg, and 5.70–20.50 mg/kg, respectively. In sediments, the TE concentrations showed narrow variations relative to riparian soils, with *CVs* ranging from 12.14% to 44.55%, suggesting that environmental disturbance to sediments is relatively minor. Yuan et al.^39^ also monitored the pollution status of sediment quality in the Jinsha River. The sediment concentrations of Cu, Zn, V, As, and Cd in the Jinsha River were of the same order of magnitude as those in the LRJR. The sediment concentrations of Zn, Cd, and Pb in our study area were lower than those in the Ganga River^36^. The sediment concentration of V in the LRJR is higher than that reported for Kharg Coral Island. The higher V concentration in the LRJR is due partly to the mining area, which has a high background value, and partly to the accumulation of V emitted from mining, beneficiation, smelting, etc., in sediment.

### Comprehensive risk assessment of TEs contamination

#### Potential ecological risk of TEs in soil and sediment

**Figure 2:**
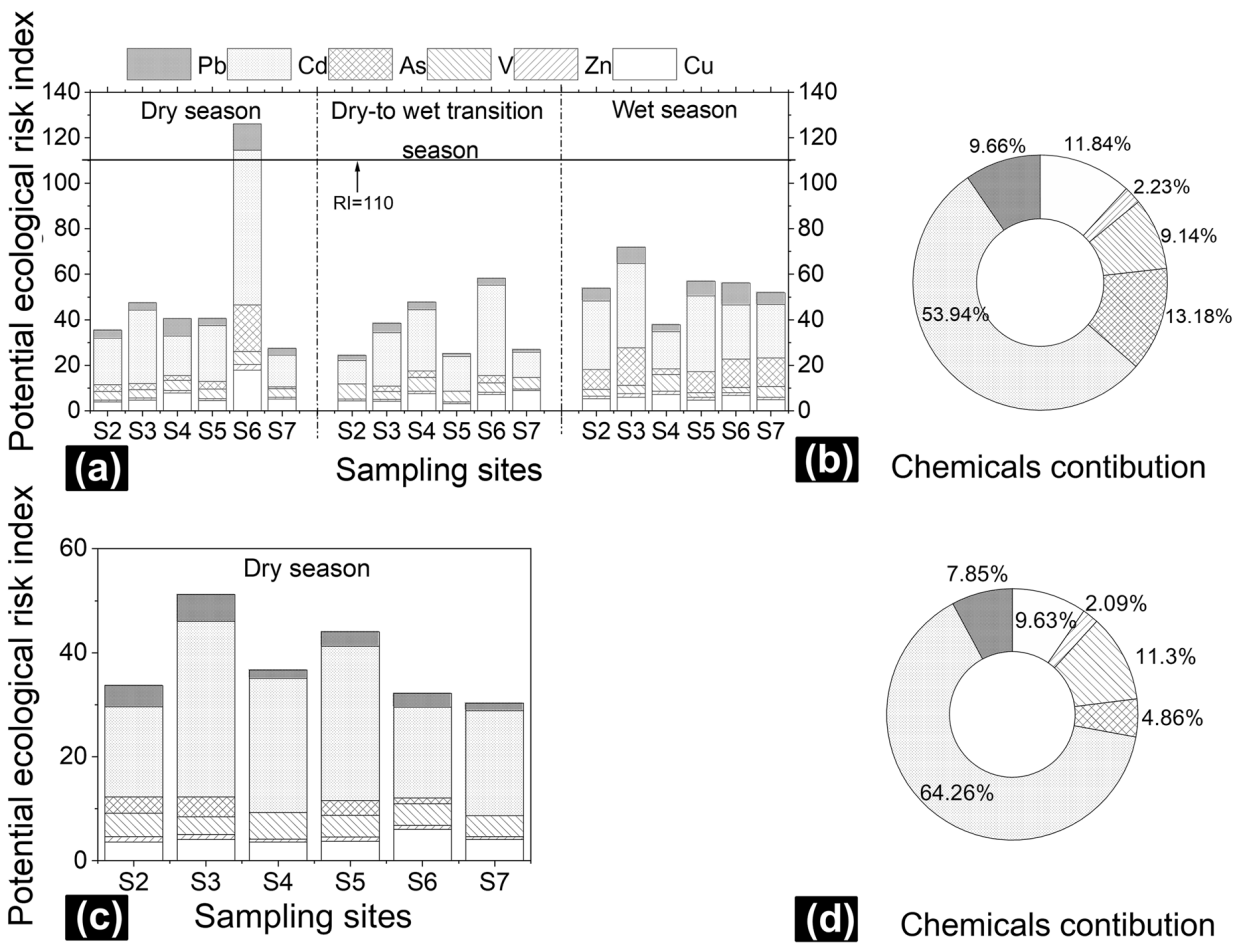
Potential ecological risk index of soil (**a**) and sediment (**c**) calculated by the Hakanson method in the study area. Each TE contribution to the index of soil (**b**) and sediment (**d**).

The potential ecological risk index values in soil and sediment calculated using the Hakanson method are displayed in Fig. 2. The *RI* values of soils ranged from 24.38 to 126.02. Only the potential ecological risk of S6 in soil during the dry season exceeded 110, suggesting that the risk reached a moderate level. This result was due to the activity of the industrial park near S6 in addition to the impact of the industrial sewage outlet. Other sampling sites were at a low risk level. Throughout the year, the contributions of each TE to the *RI* value followed the order Cd (53.94%) > As (13.18%) > Cu (11.84%) > Pb (9.66%) > V (9.14%) > Zn (2.23%) (Fig. 2b), illustrating that Cd and As were the main contributors to the soil TE ecological risk in the study area. Cadmium is a well-known environmental pollutant broadly discharged from the mining, smelting, and electroplating industries by anthropic activity. Cadmium has a high biological toxicity coefficient (with a value of 30) because it results in the overproduction of reactive oxygen species, changes in chloroplast structure, and higher lipid hydroperoxide contents in plants Wan and Zhang^45^. Thus, Cd poses a severe risk to the ecosystem. Moreover, exposure to Cd adversely affects the lungs, kidney, liver, cardiovascular system, bones, immune system, and reproductive system of humans^46^. Arsenic in the soil posed the next greatest ecological risk in this study. Human activity, including the use of As-containing pesticides, mining, and chemical production, releases As into the environment. The biological toxicity coefficient of As is 10, and inorganic As is highly toxic, causing many health problems, such as skin changes and cardiovascular and neurological disorders^47^.

The potential ecological risk index values of sediment were at a low risk level (Fig. 2c). Similar to the contributions of each TE to the *RI* in soil, Cd contributed the most (64.26%) to the *RI* in sediment, followed by V (11.30%), Cu (9.63%), Pb (7.85%), As (4.86%), and Zn (2.09%) (Fig. 2d). Moreover, the contribution of Cd in our study was higher than that in the Jinsha River (53.93%)^39^. The V contribution ranked second. The toxicity of V was smaller than those of Cd and As. However, excessive V can impact the function of the kidneys, spleen, bones, and liver^48^.

#### Human health risks posed by TEs in water and soil

**Figure 3:**
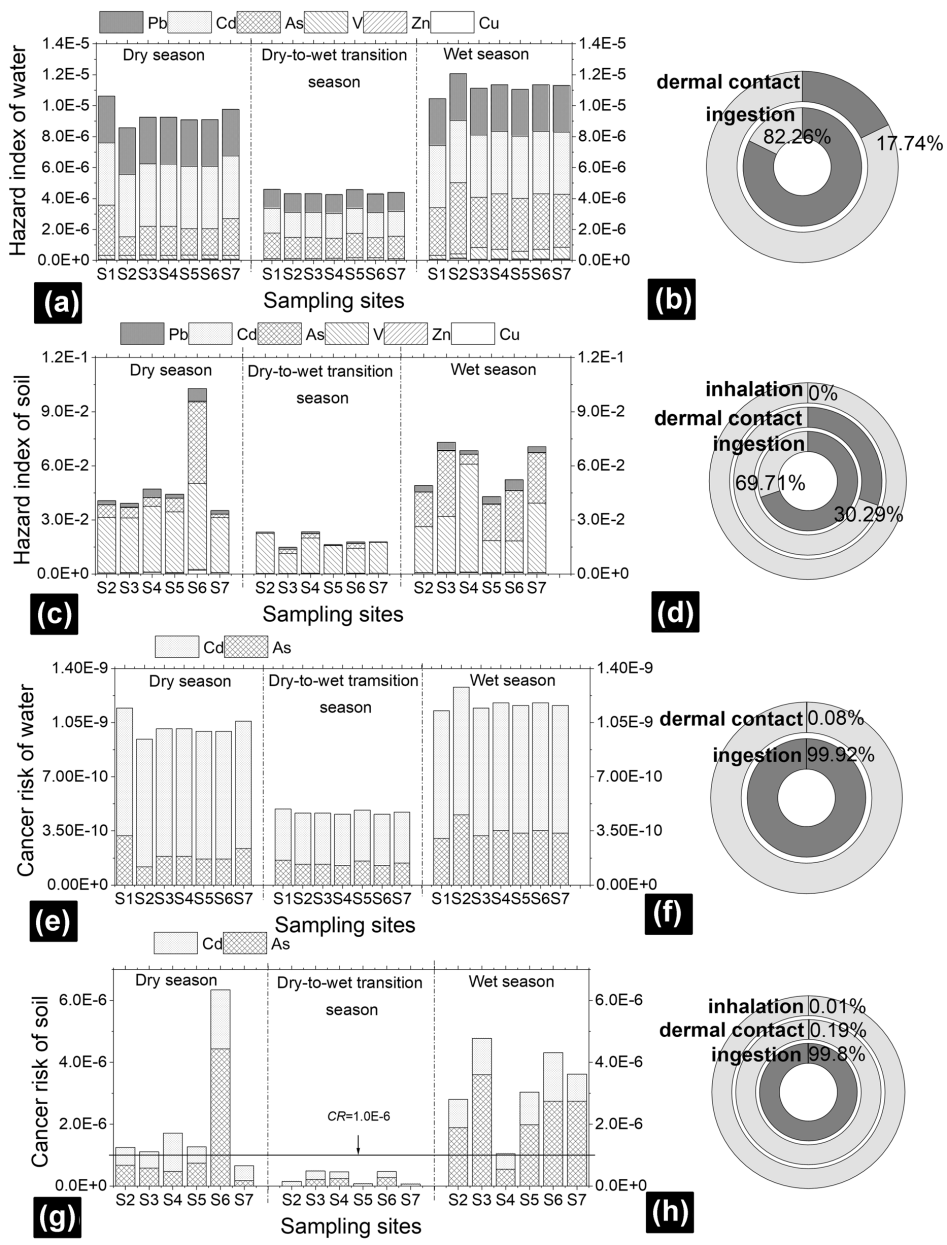
The hazard indices of water (**a**) and soil (**c**) and the contributions of exposure routes to the hazard index of water (**b**) and soil (**d**). The cancer risk of water (**e**) and soil (**g**), and the contributions of exposure routes to the cancer risk of water (**f**) and soil (**h**).

The hazard index values of water and soil at all sites were below the threshold of 1 (Fig. 3a, c), illustrating that the noncarcinogenic risk to exposed individuals was low in the study area. The *HI* value of soil ranged from 1.47 × 10^−2^ to 1.03 × 10^−1^, which was approximately ten thousand times higher than that of water (4.25 × 10^−6^ to 1.21 × 10^−5^). In surface water, the ranks of TE species that contributed to the *HI* were Cd > As ≈ Pb >  > V > Cu > Zn. The noncarcinogenic risk of TEs in water to individuals is caused predominantly by the ingestion of contaminated water (82.26%) (Fig. 3b). This is consistent with the results of Islam et al.^4^. Similar to the TE concentrations in soil, the highest noncarcinogenic risk of soil (1.03 × 10^−1^) occurred at S6 (Fig. 3c). In riparian soil, the individual TE contributions to the *HI* were ranked as follows: V >  > As > Pb > Cu >  > Zn ≈ Cd. The investigated area was contaminated by V due to the high geochemical background value and associated industrial activities^9^, especially at site S6. The soil ingestion exposure route was confirmed to be the most important, contributing 69.71% to *HI*, followed by dermal contact (30.29%) (Fig. 3d).

Only Cd and As were identified as human carcinogens, as the carcinogenic slope factors of the other TEs were unavailable^47, 49^. Therefore, the carcinogenic risks of Cd and As were calculated. The carcinogenic risks of surface water were below the limit values of 10^−6^, indicating no carcinogenic risk caused by TEs in water (Fig. 3e). Cadmium contributed 73.35% to the *CR* of water, whereas As contributed 26.65%. Similar to the *HI* of water and soil, the *CR* of soil (6.86 × 10^−8^–6.35 × 10^−6^) was higher than that of water (4.58 × 10^−10^–6.35 × 10^−9^) (Fig. 3g). The highest *CR* posed by soil TEs occurred at site S6 in the dry season. The results of each TE effect on the *CR* in soil indicated that As contributed 61.91% and Cd contributed 38.09%. In both water and soil, the effect of ingestion by gastrointestinal biota was higher for the *CR* than the effect of skin absorption (Fig. 3f, h). The carcinogenic risk posed by dermal contact and inhalation was negligible.

In general, the human health risk of riparian soil was higher than that of water. The human health risk in the dry-to-wet transition season was the lowest owing to the short exposure period (60 d/yr). For the same exposure period (150 d/yr), the human health risk in the wet season was higher than that in the dry season because of its higher concentration.

#### Regional environmental risk posed by TEs in the study area

**Figure 4:**
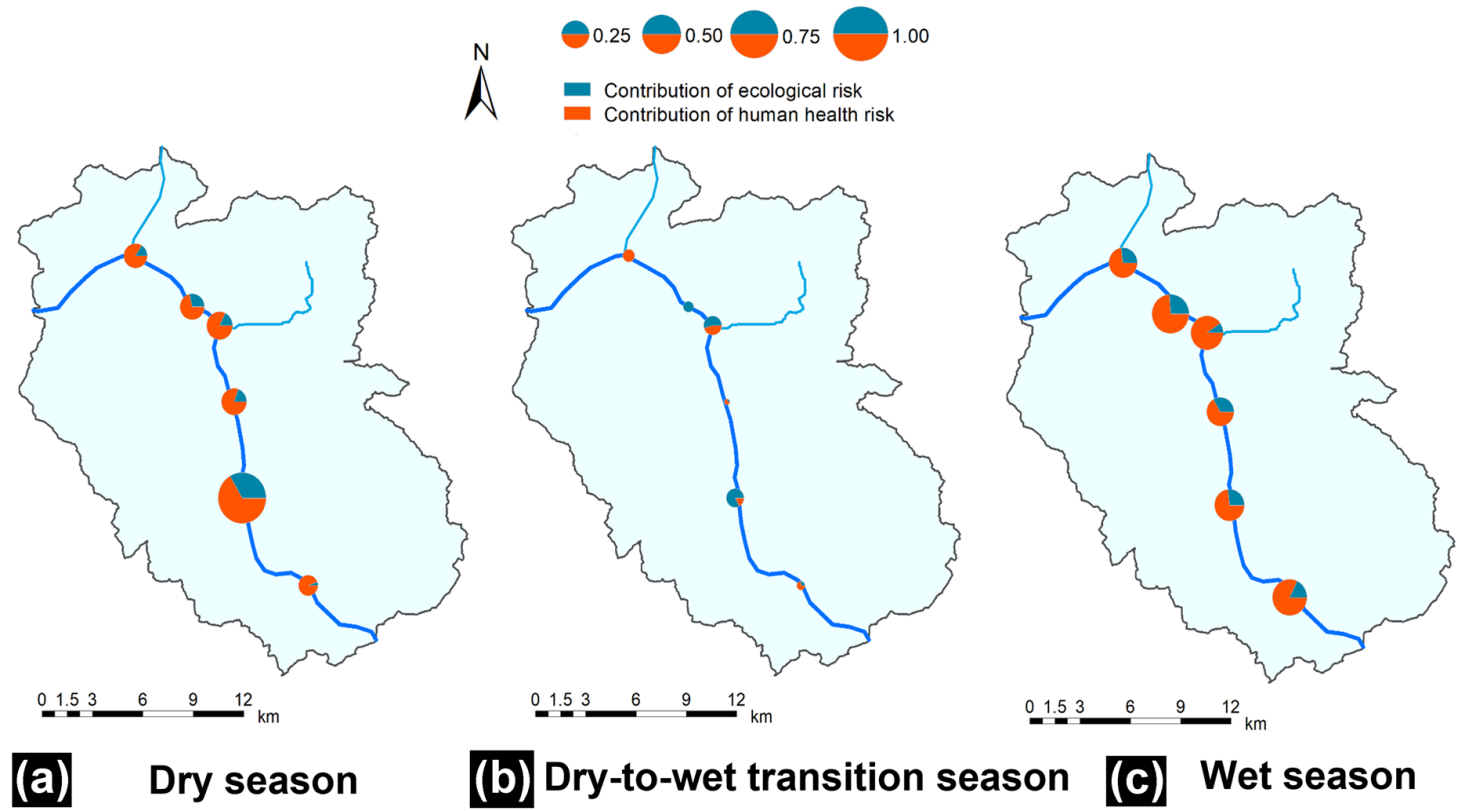
Regional environmental risk assessment results in multiple compartments in the study area. (**a**) dry season, (**b**) dry-to-wet transition season, and (**c**) wet season. This figure was generated using the ArcGIS Desktop (ESRI, Inc, Version 10.7, URL:https://desktop.arcgis.com/zh-cn/).

In this study, an analytical hierarchical process matrix (Supplementary Table S3)^50^ was applied to obtain the risk weights according to expert scoring (1–3) and effect assessment. Weights of 0.33 and 0.67 were obtained for the ecological risk and human health risk, respectively. The comprehensive assessment of regional environmental risk is shown in Fig. 4. The highest *RERI* (1.00) occurred at S6 in the dry season. This might be closely related to the intensive industrial activities and the absence of leaching and the absence of leaching during the dry season. The exhausted TE from industrial activities around S6 can accumulate in soils, and the lack of rainfall indirectly promotes TE accumulation in soils^51^. However, the average *RERI* value of the study area in the wet season (0.42) was higher than those in the dry (0.41) and dry-to-wet transition seasons (0.08) because of the combined effect of the high ecological risk posed by TEs and the long exposure time in the wet season.

The proposed *RERI* could allowed the ecological and human health risks to be assessed simultaneously. The uncertainty of our modified method was in the weights assigned to the ecological risk and human health risk. The weights should be determined by the protection target of this region. That is, the weights should be rescored under different scenarios. We also calculated the *RERI* value of our study area when the protection target shifted to the ecological environment (Supplementary Fig. S1). Obviously, the contribution of ecological risk was higher than that of the former method of assigning weights. Under this scenario, the average *RERI* values of our study area in the dry (0.37) and wet (0.36) seasons decreased, while that in the dry-to-wet season (0.11). Therefore, the weights should be a result obtained by carefully and comprehensively consulting and referencing relevant environmental policy and management research. For example, research conducted in the Atuwara River, Nigeria, which serves as a drinking water resource for over 20,000 people, was focused on the human health risk posed by TEs^21^, while studies conducted in coastal areas^8^ and river estuaries^4^ have prioritized the ecological risks of TEs.

### Multivariate analysis of TEs in multiple compartments

#### Relations between TEs in water, soil, and sediment

**Figure 5:**
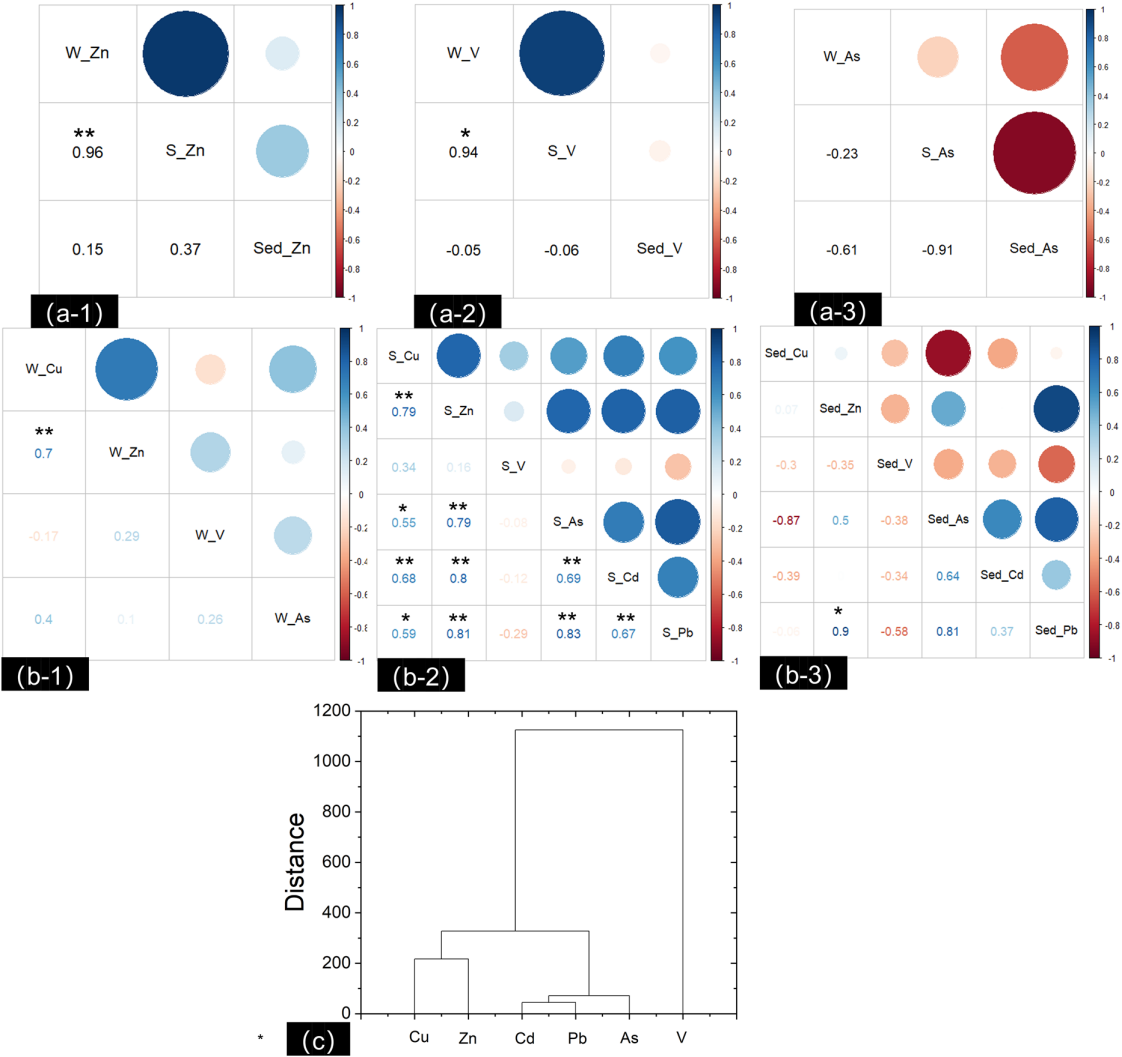
Correlations of Zn (**a-1**), V (**a-2**), and As (**a-3**) in multiple compartments and of REs in surface water (**b-1**), riparian soil (**b-2**), and sediment (**b-3**); cluster analysis of HM species in sediment (**c**). **Significant correlation at the 0.01 level (2-tailed). *Significant correlation at the 0.05 level (2-tailed). W_Zn, S_Zn, and Sed_Zn represent the concentrations of Zn in the surface water, riparian soil, and sediment, respectively.

To obtain a deeper understanding of the information on TE transport in surface water, riparian soil, and sediment, the study area should be seen as a whole system. Thus, the relationships of the Zn, V, and As concentrations in multiple media were analyzed (Fig. 5a). Zinc, which is mainly used in galvanization, is an element associated with V-minerals in the study area^9^. The relationships between the Zn concentrations in the three media were positive, meaning that Zn is a major external TE in the studied regional river ecosystem. Riparian soil and sediment both played a role as a sink of Zn, which can desorb from solid matter to water. Soil represents a large sink of V and always strongly retains V^52^. Therefore, we found that riparian soil had a high concentration of V, and we observed a significant positive relation between the soil V and water V (*r*^2^ = 0.94, *p* <  − 0.05). Arsenic in water had a negative relation with that in soil and sediment, which may be closely related to the adsorption equilibrium dynamic process of water-As versus soil-As and water-As versus sediment-As. Because of the relatively low concentration of As in environmental media, soil and sediment could still provide adsorption sites for As. Thus, adsorption–desorption processes of As occur in water–sediment and water-soil. Selim et al.^53^ found that FeOOH in sediments could adsorb As. Zinc, vanadium, and arsenic in these multiple media presented mutual influences. Unfortunately, limited research has linked the TEs in these three environmental compartments together. Gao et al.^54^ compared the TE concentrations of water, sediment, and organisms in the Bohai Sea but did not provide a quantitative relationship.

### Possible source of TE pollution in multiple compartments

The results regarding the relationship of TEs in the individual environmental media are given in Fig. 5b. Weak relations of TEs were observed in the water body, although Zn and Cu had a strong positive relationship (*r*^2^ = 0.7). A significant relation (*p* > 0.05) was found in the riparian soil, and the Pearson correlation coefficients among Cu, Zn, As, Cd, and Pb were between 0.55 and 0.83. In the sediment, Zn and Pb had a significant positive relation, with a correlation coefficient of 0.9, suggesting that these TEs may come from the same source and show interdependence and similar transport patterns^15, 21^. The relationships between V and the other TEs were not significant in these three environmental media. Similarly, Gao et al.^54^ found that V had insignificant relationships with several TEs in water, including Cu and Zn. Thus, the source of V is different from that of As, Cd, and Pb. PCA was conducted to clearly trace the possible TE sources in individual media (Table 3). One factor signified one type of possible TE pollution source. In this study, three factors with an eigenvalue above 1 or a high loading on a specific chemical were obtained, and they accounted for 98.08%, 92.21%, and 100.00% of the total variance of TEs in water, riparian soil, and sediment, respectively. The possible TE sources in water and soil were similar. Factor 1 had medium to high loadings of Cu, Zn, As, Cd, and Pb and a positive but low loading of V, which could be from industrial discharge^31^, including that from the mining and steel enterprises in the study area. Factor 2 had a high loading of V and small loadings of Cu and Zn. Based on the geological background of the study area, Factor 2 may represent a source from parental rocks^3^. Factor 3 showed a high loading of As. This result might be related to the use of As-containing fertilizer and pesticides, meaning that Factor 3 could be better explained as an agricultural source^21, 55^. Therefore, we deduced that industrial discharge, parent rock weathering, and agriculture were the three main sources of TEs in surface water and riparian soil. However, the TE sources in the sediment were slightly different from those of the other two compartments. The first component explained 55.95% of the total variance and had high loadings on As, Cd, and Pb, suggesting that these elements had some degree of homology and were mainly influenced by industrial development and agricultural activities. The weathering of crustal material associated with V was the second component of the TE source in sediment. Copper and zinc had high loadings on Factor 3, indicating that industrial sources, such as Cu–Zn alloy production, discharge TEs, which finally migrate to sediment^28^. Moreover, a cluster analysis was conducted to analyze the TE contents in sediment (Fig. 5c), and the results were consistent with the conclusion of the PCA. The TEs were divided into three groups. Detailed cluster results indicated that subclasses could be sorted within Cu, Zn, Cd, Pb, and As. Copper and zinc, as chalcophilic elements, were grouped together as a subclass based on analogous geochemical behavior in soil^56^. Cadmium, lead, and arsenic were grouped into another subclass. Therefore, we suggest that industrial discharge and agricultural activities should be regulated to protect the Jinsha River DHV, China. Additionally, as a fine particulate, dust has a high specific area upon which to adsorb TEs. Further research could focus on the risk posed by TEs in dust and biotic matrices to fill the research gap in the whole river ecosystem.

**Table 3:** Factor loadings, extraction sum of squares loadings, and percentage of HM concentrations in each environmental compartment according to the Varimax-rotated model.

Water	Component		
	Factor 1	Factor 2	Factor 3
Cu	0.873	− 0.445	0.090
Zn	0.844	− 0.073	− 0.507
V	0.278	0.915	− 0.271
As	0.575	0.340	0.739
Eigenvalues	1.882	1.157	0.885
Variance%	47.06%	28.92%	22.1%
Cumulative%	47.06%	75.98%	98.08%

Riparian soil	Component		
	Factor 1	Factor 2	Factor 3
Cu	0.451	0.278	− 0.557
Zn	0.484	0.150	0.147
V	0.121	0.826	0.256
As	0.430	− 0.318	0.664
Cd	0.427	− 0.039	0.073
Pb	0.424	− 0.341	− 0.396
Eigenvalues	3.946	1.323	0.264
Variance%	65.76%	22.06%	4.39%
Cumulative%	65.76%	87.82%	92.21%

Sediment	Component		
	F1	F2	F3
Cu	− 0.721	− 0.341	0.603
Zn	0.517	0.353	0.408
V	− 0.576	0.688	− 0.441
As	0.970	0.115	− 0.215
Cd	0.683	− 0.683	− 0.258
Pb	0.912	0.128	0.391
Eigenvalues	3.357	1.653	0.990
Variance%	55.95%	27.55%	16.50%
Cumulative%	55.95%	83.50%	100.00%

### Limitations

This study provides a comprehensive regional environmental risk assessment model and a useful method for managing the environmental quality of multiple compartments. First, a useful and comprehensive assessment method of ecological and human health risks was proposed. This allows the use of an index to intuitively show the comprehensive environmental risk of a study area. Second, we addressed the importance of monitoring the TE pollution situation in multiple compartments, which would help deepen the understanding of the TE migration process in the environment and obtain information on the whole process of TEs from their source to their receptor. This study also has obvious limitations, as does all scientific research. From the perspective of whole-process management, fine dust and biotic receptors of TEs should be considered in future research. Additionally, there is subjectivity in the weight assignment of ecological risk and human health risk, which could be improved in future work.

## Conclusions

This work investigated the TE contamination status in the LRJR, China. Ecological risks and human health risks have been assessed in water, sediment, and riparian soil, and the *RERI* method was proposed to comprehensively evaluate the overall risk. The results showed that the water quality was in accordance with the Chinese third-grade national standard. The mean concentrations of Cu, Zn, and V were 1.27, 1.07, and 2.20 times higher than the local background values, respectively. Overall, the ecological risk in the study area was at a low level, except for the riparian soil collected near an industrial sewage outlet, S6, which had a moderate risk level. The noncarcinogenic risks of TEs in riparian soil and water were ascertained to be below the thresholds, but the noncarcinogenic risk of V in soil should be evaluated further. While the carcinogenic risk was significant, the probability of cancer occurring due to exposure to TEs in soil was 1 out of 1.57 × 10^5^–10^6^ people. Ingestion via the gastrointestinal tract contributed the most to the human health risk, followed by dermal contact and inhalation. The *RERI* value was higher in the wet season than in the dry-to-wet transition and dry seasons, and the proportion of ecological risk and human health risk varied. Source apportionment analysis suggested that industrial discharge, weathering of parent rocks rich in V, and agricultural pollution were the possible sources of TE contamination. Measures should be implemented in the whole study area to reduce agricultural discharge, and additional measures should be conducted at site S6 to restrict industrial effluent. This study provides a useful method for managing the environmental quality of multiple compartments and a comprehensive regional environmental risk assessment model. Future studies could extend the multiple-compartment approach to include the atmosphere and biotic matrices.

## Supplementary Information

Supplementary Information.

## Abbreviations (Excluding those that are used only in equations)

LRJR: Lower reach of the Jinsha River
TE: Trace element
DHV: Dry-hot valley
*RI*: Potential ecological risk index
*HI*: Hazard index
*CR*: Cancer risk
*RERI*: Regional environmental risk index
PCA: Principal component analysi
