# Supplementary material for: Risk assessment and source apportionment of trace elements in multiple compartments in the lower reach of the Jinsha River, China
Source: Sci Rep. 2021 Oct 8;11:20041. doi: 10.1038/s41598-021-99626-w (PMC8501140; doi:10.1038/s41598-021-99626-w)
Supplement: Supplementary file 1 — Supplementary Information. [file 41598_2021_99626_MOESM1_ESM.pdf]

Supplementary Information

Risk assessment and source apportionment of trace elements in multiple compartments in the lower reach of the Jinsha River, China

Wenyan He[a], Fei Li[b], Jiang Yu[c], Min Chen[a]*, Yun Deng[a], Jia Li[a], Xiliang Tang[c], Zhuoyu Chen[a], Zhongluan Yan[c]

[a] State Key Laboratory of Hydraulics and Mountain River Engineering, College of Water Resource & Hydropower, Sichuan University, Chengdu, 610065, China

[b] Changjiang Water Resources Protection Institute, Wuhan, 430051, China

[c] China Three Gorges Projects Development Co., Ltd, Chengdu, 610000, China

* Correspondence: No. 24 South Section 1, Yihuan Road, Chengdu, 610065, China; Email: mchen@scu.edu.cn (Dr. Min Chen)

Figures

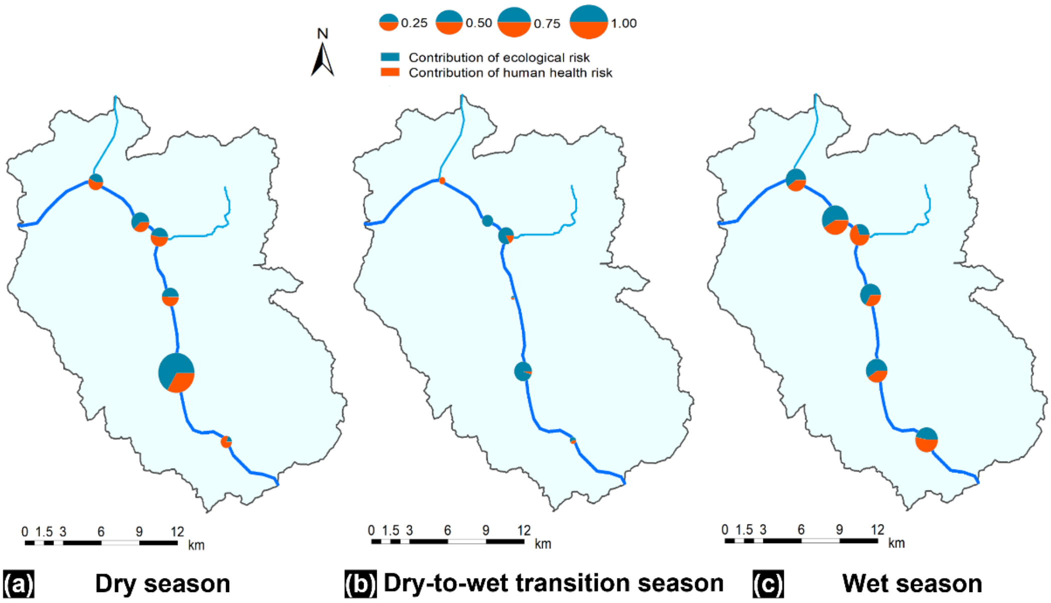

Fig. S1. Regional environmental risk assessment results in multiple compartments of the study area, in which the weights of ecological risk and human health risk were 0.67 and 0.33, respectively. This figure was generated using the ArcGIS Desktop (ESRI, Inc, Version 10.7, URL:https://desktop.arcgis.com/zh-cn/).

Tables

Table S1 Hierarchical classes of potential ecological risk according to RI_i and RI[1].

RI_i	RI	Potential Ecological Risk Grade
$RI_i < 40$	$RI < 110$	Low
$40 \leq RI_i < 80$	$110 \leq RI < 220$	Moderate
$80 \leq RI_i < 160$	$220 \leq RI < 440$	Considerable
$160 \leq RI_i < 320$	$440 \leq RI < 880$	High
$RI_i \geq 320$	$RI \geq 880$	Severe

Table S2 *RfD* and *SF* values of different exposure pathways of TEs[2].

Element	RfD_{ing}	RfD_{derm}	RfD_{inh}	SF_{ing}	SF_{derm}	SF_{inh}
Cu	0.04	0.012	0.04	–	–	–
Zn	0.3	0.06	0.3	–	–	–
V	0.007	0.00007	–	–	–	–
As	0.0003	0.000123	0.3	1.5	3.66	15.1
Cd	0.001	0.0000125	0.001	6.1	–	6.3
Pb	0.0035	0.0005250	0.00352	–	–	–

Table S3 Analytical hierarchical process weight matrix of ecological risk and human health risk.

	Ecological risk	Human health risk
Ecological risk	1	0.5
Human health risk	2	1

References

1 Islam, M. S., Hossain, M. B., Matin, A. & Sarker, M. S. I. Assessment of heavy metal pollution, distribution and source apportionment in the sediment from Feni River estuary, Bangladesh. *Chemosphere* **202**, 25-32, doi:https://doi.org/10.1016/j.chemosphere.2018.03.077 (2018).

2 Ferreira-Baptista, L. & De Miguel, E. Geochemistry and risk assessment of street dust in Luanda, Angola: A tropical urban environment. *Atmospheric Environment* **39**, 4501-4512, doi:https://doi.org/10.1016/j.atmosenv.2005.03.026 (2005).
